# An Optimized Hyperparameter of Convolutional Neural Network Algorithm for Bug Severity Prediction in Alzheimer's-Based IoT System

**DOI:** 10.1155/2022/7210928

**Published:** 2022-06-28

**Authors:** Iqra Yousaf, Fareeha Anwar, Salma Imtiaz, Ahmad S. Almadhor, Farruh Ishmanov, Sung Won Kim

**Affiliations:** ^1^Department of Computer Science and Software Engineering, International Islamic University, Islamabad 44000, Pakistan; ^2^College of Computer and Information Sciences, Jouf University, Sakaka, Saudi Arabia; ^3^Department of Electronics and Communication Engineering, Kwangwoon University, Seoul 01897, Republic of Korea; ^4^Department of Information and Communication Engineering, Yeungnam University, Gyeongsan 38541, Republic of Korea

## Abstract

Softwares are involved in all aspects of healthcare, such as booking appointments to software systems that are used for treatment and care of patients. Many vendors and consultants develop high quality software healthcare systems such as hospital management systems, medical electronic systems, and middle-ware softwares in medical devices. Internet of Things (IoT) medical devices are gaining attention and facilitate the people with new technology. The health condition of the patients are monitored by the IoT devices using sensors, specifically brain diseases such as Alzheimer, Parkinson's, and Traumatic brain injury. Embedded software is present in IoT medical devices and the complexity of software increases day-by-day with the increase in the number and complexity of bugs in the devices. Bugs present in IoT medical devices can have severe consequences such as inaccurate records, circulatory suffering, and death in some cases along with delay in handling patients. There is a need to predict the impact of bugs (severe or nonsevere), especially in case of IoT medical devices due to their critical nature. This research proposes a hybrid bug severity prediction model using convolution neural network (CNN) and Harris Hawk optimization (HHO) based on an optimized hyperparameter of CNN with HHO. The dataset is created, that consists of the bugs present in healthcare systems and IoT medical devices, which is used for evaluation of the proposed model. A preprocessing technique on textual dataset is applied along with a feature extraction technique for CNN embedding layer. In HHO, we define the hyperparameter values of “Batch Size, Learning Rate, Activation Function, Optimizer Parameters, and Kernel Initializers,” before training the model. Hybrid model CNN-HHO is applied, and a 10-fold cross validation is performed for evaluation. Results indicate an accuracy of 96.21% with the proposed model.

## 1. Introduction

Software is part of our daily life, present in almost all domains such as education, health, business, and manufacturing. Software engineering is the main discipline in which high quality software is developed by applying user-centered design principles. Different tasks and operations of an organization are handled by software systems [1]. The new IoT technology is gaining attention with these healthcare systems for medical filed, and IoT devices such as sensors and smart equipment are using for the treatment of the patients [2, 3]. Multiple software used in IoT medical devices for remotely working and complexity of software increases day by day. With the increase in size and complexity of software, the number of reported bugs becomes huge. These bugs in IoT medical devices and other healthcare systems can have severe consequences if there is malfunction. The bug in IoT medical devices such as for Alzheimer's patient is like swallowable misinformation, means doctor checks the compliance of a patient with an Alzheimer's disease and recommend him pills with a swallowable chip. However, due to a bug in the device's design, the transmitter is unable to communicate compliance data to the physician. The doctor is unaware that the patient is not taking his medication, leading the situation to worsen [4]. A software bug is defined as a fault or defect in the program. These faults or defects can occur during different phases of software development life cycle, i.e., coding, designing, or maintenance. The timely resolution of these faults is critical to project success. Bug can occur due to any reason such as error by a developer, misunderstanding or inaccurate execution of requirements, and inadequate communication between team members or with users [5] (Table 1).

In software development life cycle, the most significant part of software development is software maintenance with respect to bugs. Defect prediction techniques are used in software maintenance process to improve the reliability of software by isolating a bug. The main focus of software maintenance is to resolve the bug reported by users and testers of the system when it is tested or used [1, 6]. In software development life cycle, 90% software's cost is spent during the phase of software maintenance. Research community focuses on bug prediction for software maintenance and evolution because of the huge number of bugs. In bug fixing process, a bug is first prioritized on the basis of the severity [7] There is a difference between severity and priority: severity is the level of impact on the performance of the software system, whereas priority is the order in which the bug is resolved. Priority of a bug depends on the severity level [8]. Thus, the severity of a bug has significant importance for bug prioritization and resolution. The fields of a bug report are considered as the features of prediction model. The fields are Bug Report ID, Created Date, Project Name, Priority, Severity, Summary, Component and Description of Bug, Assignee, Reporter, etc [9].

Earlier, defect prediction techniques that are used in medical devices are statistical analysis tool; fault localization and model checking are used to identify the defect in a software [10–12]. Research is focusing on the use of machine learning techniques to identify the bugs in software. Bug severity can also be assigned using ML algorithms. Literature has defined multiple levels of severity such as severe and nonsevere [6, 13], blocker, critical, major, minor, and trivial along with addition of normal and enhancement level [14]. User assigns the level of severity when they report the bug, and it causes an inappropriate assessment of severity due to inexperienced user and lack of domain knowledge. The manual severity level assignment is a very time-consuming, erroneous, and difficult task [6]. It depends on the domain knowledge and experience of users. Machine learning approaches are introduced to overcome the limitations of manual severity level assignment.

*Motivation*: Machine learning approaches are introduced to overcome the burden of manually assigning severity levels. The multiple ML approaches such as ensemble techniques, comparison of different algorithms such as NB, RF, KNN, MLR, J48, and RNG, CNN and SMOTE for severity assignment is performed [1, 15, 16]. However, these approaches do not consider proper feature selection methods [17] and emotion-based technique [6] and so on. By considering these limitations, researchers have proposed deep learning (DL) approaches for the bug severity prediction. DL is a subset of ML, which is capable of self-learning from the data. Different approaches such as K-NN, CNN, RNN-LSTM, CNRFB model, and CMT with weights are proposed [6, 15, 16]. These techniques also have some drawbacks, such as they do not consider large datasets, unlabeled datasets, and imbalanced datasets. They also do not consider important parameters known as hyperparameters in deep algorithms [6], hence compromising accuracy. Parameters are those whose values are updated during training by some optimization tool for ML classifiers. Hyperparameters are those parameters whose values are decided before training the model, and these hyperparameters are optimized for ML or DL classifier to enhance their performance. Hyperparameter optimization is the method of optimizing hyperparameters of different classifiers, and we can relate these parameters to model selection such model type, model architecture, or learning algorithm [18].

In IoT medical devices, when a new bug is found, developers, users, and testers have to assign a severity level to it. Since lack of attention to critical bugs can result in severe injury or even death of a patient. Many automatic techniques for bug severity prediction are introduced by using ML algorithms and DL algorithms in general softwares; however, no work is performed on a healthcare IoT medical dataset. The need for accurate prediction of bug severity in a timely manner is critical to its timely resolution. The aim of this research is to identify an optimized hyperparameter of the CNN algorithm by using the HHO algorithm for bug severity prediction in healthcare dataset that improves accuracy. The research contributions are given one by one:

- A novel optimized hyperparameter technique of convolutional neural network for bug severity prediction
- Better accuracy of bug severity prediction with the help of proposed technique
- Early detection of bugs in IoT medical devices especially for Alzheimer disease
- Proposed technique that provides better fitness value (WSM)

The paper is organized in sections as follows.  Section 2 describes the literature review of techniques for bug severity prediction, Section 3 describes the research design of proposed work, and the results, finding, and discussion are presented in Section 4.

## 2. Literature Review

In this section, we divide and present the literature on the basis of healthcare medical devices and general softwares' on severity level prediction during software maintenance.

### 2.1. Defect Prediction Techniques Used for Software Medical Devices

*Medical devices:* they mostly rely on embedded software. Due to the critical nature of the health domain, the medical devices must be defect free. Therefore, the manufactures perform detail verification and validation of the embedded software in them. Different defect prediction techniques such as statistical analysis, fault localization, code review, metrics, and model checking are used in medical devices. [10–12]. Statistical analysis is used to check a software system without executing the software. Modern analysis tools help in reducing software cost by detecting the defects earlier in software development life cycle [10].

### 2.2. Machine Learning Approaches in Healthcare

 
*Machine learning (ML) approaches*: they help in providing promising opportunities for improving the delivery of quality health care [19, 20]. Currently, different ML approaches are used for analysis of the big healthcare data [2]. They are used in identification and diagnosis of disease, drug discovery and manufacturing personalized medicines, pattern imaging analysis, smart-health record, crowd-sourcing data collection, and clinical trial research [21]. The use of ML algorithms is gaining attention in healthcare; however. ML is not used for severity level prediction. A detailed review is provided about machine learning algorithms that are applied on various healthcare big data [22]. ML approaches such as supervised learning, unsupervised learning, and reinforcement learning are used on healthcare big data included in the electronic medical records, medical imaging, Internet of things, medication, etc.
 
*Support Vector Machine*: A hybrid technique for software defect prediction in medical software is proposed. SVM parameters are optimized with the genetic algorithm. Experiments are performed to check defect prediction. Results show better performance as compared to other state-of-the-art techniques [23].
 
*Convolutional Neural network*: Computer-aided design, convolutional neural network technique proposed for diagnosing brain tumor to improve the diagnosis accuracy. BR35H benchmark dataset is trained that consists of brain tumor MRIs. Six-different datasets used for evaluate the model performance and to enhance the performance different geometric data augmentation techniques, with statistical standardization are selected. The proposed system performed better with average 98% accuracy and around 0.99 specificity by comparing other systems [24]. In [25], Gaussian convolutional neural network (GCNN) was proposed on two datasets to detecting distinctive brain tumor types. Tumors classify into pituitary, glioma, and meningioma in one dataset and other dataset divided into three grades of glioma. Accuracy achieves with two datasets 99.8% and 97.14% on proposed approach, respectively.

### 2.3. Bugs in Healthcare Medical Devices

The consequences of software failures are huge in case of healthcare. Software-related failures in medical devices cause severe injuries or death and should be resolved on time. The proposed technique provides the analysis of software related system failures of medical devices. System failures are categorized by their symptoms and faults and different methods for preventing and detecting faults. The nature of fault helps in identification of prevention and detection strategy before the system is released. This technique also provides the detailed insight about the formal requirements specification and improves the testing of the complex systems [26]. Thomas provided 14 specific suggestions for early detection of computer-related bugs in medical devices before detected by clinicians, procurement, and regulators. They also describe different ways to reduce severity of bugs. These suggestions help to avoid bugs with safer use and improved quality of healthcare systems, as a result saving lives of patients and money [27].

### 2.4. IoT-Based Healthcare Support Systems

IoT devices are the new technology that used for facilitating and helping the people. Many sensors and devices with applications are used for monitoring the health condition of patients in their home. Alzhemier's patient treat on the basis of their behaviours and movement and data gathered from the sensors and equipment installed at patient's home. Different types of protocols are used for the sensor and smart equipment [3]. The Uk HMG ISI technique is proposed for threat analysis in Medical Internet of Things (MIoT) systems, and the case study is performed in the form of Technology Integrated Health Management test bed. The complete threat's assessment is conducted by determining the static or dynamic features in the component of MIoT systems. These features have an impact on the dependencies and space threats when updating the system. The proposed technique saves time and effort when identifying the threats in MIoT systems. The MIoT system is a healthcare system that monitors the devices and tracks the condition of the patient remotely by recording the specific health measurements systemically and send the complete information to back-end system. The system examines the collected data and detects the health issues of patients earlier for any emergency [28].

### 2.5. Machine Learning for Bug Severity Prediction for General Software

 
*Ensemble Techniques:* an ensemble method for detection of bugs on NASA project PITS A-F dataset is proposed by using Bagging, Voting, AdaBoost, and Random Forest. Results indicate that better accuracy is achieved with the help of bagging [15]. Kumari et al. applied different ML techniques on PITS Project A-F closed source dataset and opensource Mozilla dataset. KNN, J48, RF, RNG, NB, CNN, and MLR for the prediction of bug severity. MLR has highest accuracy i.e., 98.90% of NASA pits *D* and 80.37% for Eclipse and J48 was best for Mozilla 75.71% [29]. XGBoost: this used in Bugzilla repository of Mozilla Project [30]. Summary features are used in first case; summary, priority, and component are used in second case; in third case summary with SMOTE. Average accuracy achieved is 72.99%, 73.87%, and 62.23%.
 
*K-NN and SVM:* K-NN classifier was used with distance-weighted voting scheme for the prediction of severity of bug on Eclipse and Mozilla projects dataset. F-measure value for some classes increased from 34.4% to 46.6% which is higher than some other approaches (44.47%) and for some classes decreased by comparing different approaches (53%) [16]. Kumar et al. [31], applied eleven machine learning algorithms on Mozilla and Eclipse projects. SVM with kernel performed better from all others. Classification and regression techniques were used to examine the link between bug attribute. Multiattribute-centered classification and regression model is proposed for the prediction of severity and bug fix time. Sharma et al. [32] used real world datasets namely Bugzilla, Firefox, Boot2Gecko, Webtools, Thunderbird, Firefox, Seamonkey, Add-OnSDK, and Mozilla for bug severity prediction. Summary weight and bug age attribute are considered to be good predictor. Baarah et al. [33] used eight classifiers and compared their results on closed source projects dataset. Highest accuracy achieved by LMT is 86.31%, AUC 0.90%, and F-measure 0.91%.
 
*Naïve Bayes:* NB classifier is used for bug severity prediction with PSO and ACO feature extraction techniques on Mozilla, Firefox, and Eclipse datasets. Good results are achieved for precision recall and F-measure [34]. Emotion similarity multinomial NB classifier is applied on Eclipse, GNU, JBoss, Mozilla, and Wireshark with average accuracy of 70.86%, 80.32%, 88.12%, 55.65%, and 41.63% with respective datasets [35].
 
*Convolutional Neural network:* CNN algorithm applied on seven products of Mozilla and Eclipse. Average performance of proposed approach in terms of accuracy, precision, recall, F-measure are 88.10%, 82.64%, 86.16%, 84.36%, and 0.286 [6]. CNN is used with genetic algorithm (feature extraction) on Mozilla and Eclipse. Average results in terms of precision 77.38%, F1-Score 68.76%, and Recall 62.09% [36]. Multiaspect feature approach is proposed for feature extraction on Eclipse and Mozilla dataset that feed into the convolutional neural network algorithm. Average results are accuracy 75%, precision 78%, F1-measure 86%, and MCC 41%, respectively [13]. For multiclass severity classification, BCR approach is proposed based on CNN and RF with Boosting [6]. They used Mozilla, Eclipse, JBoss, OpenFOAM, and Firefox datasets with three attributes [17]. XGBoost [30], CNN, and RNN are applied on a NASA'PITS dataset for severity prediction. Highest accuracy achieved by CNN 79% in terms of AUC and sensitivity with value 0.92 and 76.34% [37]. In [38], Hamza proposed a framework where comparison of RNN [37] and LSTM is done. They collected a dataset from a closed-source project from JIRA repository. LSTM achieve higher accuracy of 85%.

Bug severity predication is of significant importance for timely resolution of critical bugs. Many ML techniques are discussed for bug severity prediction in general softwares. Timely detection and resolution of healthcare bugs is of critical importance. Machine learning is gaining attention and some studies have discussed ML models for healthcare problems in terms of diseases and medical software devices for removing software faults. Many studies have used ML for preventing and detecting software faults, but no work is done on bug severity prediction. Bugs in healthcare applications and IoT devices cause severe effect, such as Therac-25, massively overdosed due to bug, interruption in a software function such as loss of correct functions over several upgrades, inaccurate health records, circulatory suffering, and swallowable misinformation. Therefore, the significance of bug severity prediction of healthcare data is huge.

## 3. Proposed Methodology

**Figure 1:**
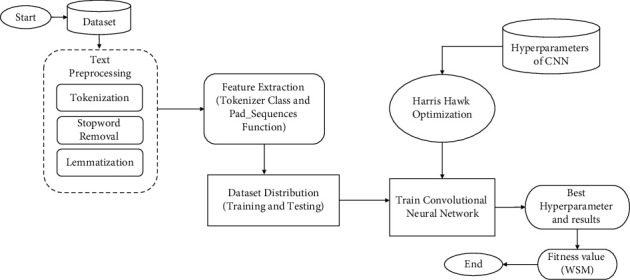
Proposed approach of CNN-HHO.

The proposed model predicts the bug severity level on the basis of summary of bug report dataset. The validation of the proposed approach is done by conducting a controlled experiment, and accuracy, precision, recall, and f1-measure is calculated. Controlled experiment is conducted on python language. In experiment, the independent variable is tested and adjusted to determine its impact on the dependent variable. In this research, independent variables (optimized hyperparameter) are used to test the impact of independent variable on dependent variable i.e., accuracy of the proposed model. The proposed model consists of different steps, the first step is to select the dataset. The second step is to perform the date preprocessing that consists of three techniques namely as tokenization, stopwords removal, and stemming. Afterwards, data is split into training and testing parts by applying 10-fold cross validation. The next step is feature extraction for embedding layer in the CNN model and the last step is to perform experiment where we apply CNN with HHO for optimizing hyperparameters of the model. Finally, the model provides the experimental results. The proposed approach is explained in Figure 1.

### 3.1. Experimental Setup

The experiment is conducted using PYTHON to answer the research question.

**RQ1**: What is the impact of the proposed approach of optimizing hyperparameter of the convolutional neural network algorithm on the accuracy of bug severity prediction?

### 3.2. Experimental Dataset

In this study, bug report is created of healthcare domain by considering bugs in different medical applications such as Therac-25 [39], from articles related to software faults in medical software devices [26, 27] and from article related to IoT-based healthcare systems for cognitive disease such as Alzheimer [4] and from article related threat's assessment in IoT medical devices [28]. The model uses these attributes from the bug report dataset i.e., bug ID, summary, and severity levels of the bug. In general softwares, bug report dataset consists of bug with the following severity levels normal, minor, trivial, enhancement, blocker, critical, and major. Normal severity level is not considered in this work due to the nature of healthcare domain. We have categorized minor, trivial, and enhancement as nonsevere bugs, while blocker, critical, and major as severe bugs. Therefore, the bugs are categorized as severe or nonsevere i.e., binary classification.

### 3.3. Bug Report Preprocessing

Preprocessing steps are performed to eliminate the unnecessary words from the bug report dataset summary [17]. Data preprocessing step is the main step to achieve more accurate features from the summary. Moreover, the classification accuracy of the prediction model is increased by using preprocessed data. For applying natural language processing methods, we use natural language toolkit (NLTK). [40]. One example of bug report preprocessing is shown in Table 2.

 
*Tokenization:* Tokenization is a process in which large string of a text data is converted into words and these words are known as tokens. In bug report, there are textual unstructured data, unrelated symbols, and punctuation marks, i.e., “!”#$%&∖”() ^*∗*^+, −./:;?@[\\]∧ _“{*|*}∼.” Tokenization first remove these punctuations and symbols, convert remaining meaningful words into tokens.
 
*Stop-word Removal:* Stopword removal is a process in which the words or terms that we use to make a simple sentences in English language are removed i.e. nouns, prepositions (“i,” “me,” “my,” “myself,” “we,” “our,” “ours,” “ourselves,” “you,” “you're”) are called as stopwords. All these words are removed in this step because these words can complicate the prediction model. The dimensionality of data becomes high, and classification efficiency of ML algorithm becomes low. The process has two parts; first we apply NLTK Library for mining the stopwords from data. In second steps, we remove all these words from data.
 
*Lemmatization:* Lemmatization is a procedure in which context of a dataset is seen and each word is converted into meaningful dictionary form. These words are known as lemma. For example, when we apply the lemmatization process, the word “selected” is converted into the basic word “select.” The last step is lemmatization; assuming Ln as the number of preprocessed words into tokens.

### 3.4. Dataset Distribution

The dataset is divided into two parts, training and testing dataset by using 10-fold cross validation. The 10-fold cross validation is applied on over all document level to protect the model from overfitting. In 10-fold cross validation, the training data is divided into 10 data subsets that are of almost same size and then the testing take place in 10 iterations; in each iteration, the one fold which contains the 10% of the dataset is used for testing, and other nine fold which contains 90% of the datasets is used for training. By this way, each data sample is used once in both testing and training. The objective of using the 10-fold cross validation is to reduce the chances of biasness and to achieve the best performance result [41].

### 3.5. Feature Extraction

Feature extraction is also called as word to vector representation. The next step is to extract a feature from the preprocessed textual dataset, and for this, we encode dataset as a sequence of integers by using tokenizer class in Keras API. Vocabulary size of all tokens can be determined by mapping words in the vocabulary to unique integers. In a neural network algorithm, the input must have the same shape and size. When we use the textual data for the neural network model as inputs, then all the sentences in the dataset do not have the same length. Naturally, some sentences have a shorter length and some have a longer length. For this, we need padding of the same size as the input for the neural network model [42]. To ensure that every statement in the dataset has same length, all statements are padded to make them equal to the length of the longest statement in the dataset. The longest statement length in the dataset is 13. Keras function Pad_Sequences are used to pad the sequences to the maximum length by using 0. In the neural network model, embedding layer is used as a first hidden layer and it must have three arguments. These arguments are input_dim (vocabulary size of dataset i.e., if integer encoded value of dataset is 0–10, then vocabulary size is 11), output_dim (the size of the real-valued vector space in which the word is embedded, and for the embedding layer, the size of the output vector is defined for each word, and embedding dimension size is defined for output vectors 50), and input_length (maximum length of input dataset), these are the arguments required for embedding layer. The research has used small dataset for the problem, so the embedding dimension size used is 50. Different embedding dimension values can be tested according to the problem [43].

### 3.6. Deep Learning and Convolutional Neural Network

DL has many hidden layers in contrast to ML. ML as well as DL both can be supervised and unsupervised learning. The benefit of considering DL over ML is that there is no need of data preprocessing for numerical dataset and feature selection [44]. Many benefits of DL make it suitable to adapt it for new problems. Convolutional neural network is a DL approach with many benefits [45].

**Figure 2:**
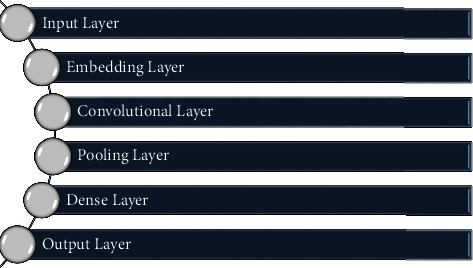
Simple architecture of CNN.

Convolutional layer is always considered as a first layer in a hidden layers and last layer is always considered as fully-connected layer. In the convolutional layer, we gather the related feature from the input layer of the data and output of this layer passes through an activation function. The output of the neural network is calculated by using the activation function. There are many activation functions, and the use of activation function depends upon the define problem. Activation functions are categorized into two types, namely linear and nonlinear. The pooling layer merges all the same features into one pool. Pooling layer has many advantages; it decreases the dimensionality, and when used after every convolutional layer, it decreases the computational complexity and also helpful in overfitting problem. Pooling layer has many types namely max, average and, sum. They are used according to the define problem. Fully connected layers use to transform the input data in N-dimensional vectors. Here, N is defined as number of classes or label that is used to classify the target data. CNN is the simply feed-forward neural network. In some problems, dropout layer is used and it is very helpful to overcome the overfitting problems of networks. Parameter optimizer plays a main role in calculating the performance of CNN [46]. The working of layers of CNN is expressed in Figure 2.

### 3.7. Harris Hawk Optimization Algorithm

**Figure 3:**
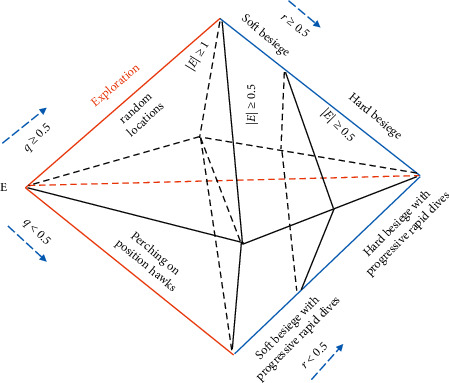
Exploration and exploitation phases [46].

The Harris Hawk Optimization (HHO) Algorithm is a population-based swarm technique introduced by Heidari et al. [47], used to solve the optimization problem. HHO is inspired by the Harris Hawks chasing style, and their cooperative behavior toward prey in nature known as surprise pounce. This technique explains the overall performance of hawks in mathematical form, such as how they cooperate to search for the prey, hunt, surprise, and chase it from different directions. These behaviors of Harris Hawks are used to develop an optimization algorithm for solving complex problems. The HHO consists of both phases i.e., different optimization techniques and, exploration and exploitation. The summary of two stages of HHO (exploration and exploitation) is present in Figure 3.

#### 3.7.1. Exploration Stage

First step of Harris Hawks is exploration phase, by considering nature where Harris Hawks are not able to detect the prey properly, they wait, search, and explore for the desired prey for several hours. In Harris Hawk optimization technique, candidate solutions are the Harris Hawks, and they consider the best candidate solution by perching on some positions and wait to identify a prey by using two strategies (exploration and exploitation). These strategies selected on the basis of probability and the probability are defined as *p*. In first strategy, when *p* value is *p*〈0.5 , the Harris Hawks perch on the basis of the position of prey and the other hawks and in second strategy when *p* ≥ 0.5 , the Harris hawks perch randomly on the tall trees with random location in a specific range. These two strategies are modeled in equation (1). The detail description of equation (1) is described in Table 3.

(1) $$\begin{array}{c} Z\left(t+1\right.=\left\{\begin{array}{cc} Z_{\text{rand}}\left(t\right)-r_{1}\times \left|Z_{\text{rand}}\left(t\right)-2\times r_{2}Z\left(t\right)\right| & \text{if }p\geq 0.5\\ \left(Z_{\text{prey}}\left(t\right)-Z_{m}\left(t\right)\right)-r_{3}\left(L_{B}+r_{4}\left(U_{B}-L_{B}\right)\right) & \text{if }p<\;\langle )0.5 \end{array}\right). \end{array}$$

Average location of the hawks is evaluated by

(2) $$\begin{array}{c} Z_{m}\left(t\right)=\frac{1}{N}\sum_{i=1}^{N}Z_{i}\left(t\right), \end{array}$$

where *Z*_*i*_(*t*) is defined as the current position of the hawk in the iteration *t* and *N* as the whole numbers of hawks in the population. Average location can be obtained in many ways but the easiest method is considered.

#### 3.7.2. Transition from Exploration to Exploitation

There is another stage known as the “transition from exploration to exploitation” in which on the basis of prey's escaping energy they calculate the change between different exploitative activities. In this stage, prey stabs to escape from Harris Hawks and prey's escaping energy decreasing. The escaping energy of the prey is evaluated by

(3) $$\begin{array}{c} E=2\times E_{o}\times \left(1-\frac{t}{T}\right). \end{array}$$

Prey's escaping energy is defined as *E*, initial energy of the prey indicate as *E*_*o*_, and *T* is the total number of iterations. *E*_*o*_ Initial energy of the prey lies between the interval (−1, 1); when *E*_*o*_ the value reduces from 0 to −1, then prey is actually flagging and when *E*_*o*_ value enhance from 0 to 1, then prey has power of escaping. During iterations, *E* dynamic escaping energy continuously reduces. The dynamic escaping energy *E* indicates that the exploration stage has not finished; when |*E*| ≥ 1, then exploration phase occurs, while |*E*|〈1, then exploitation stage occurs.

#### 3.7.3. Exploitation Stage

**Figure 4:**
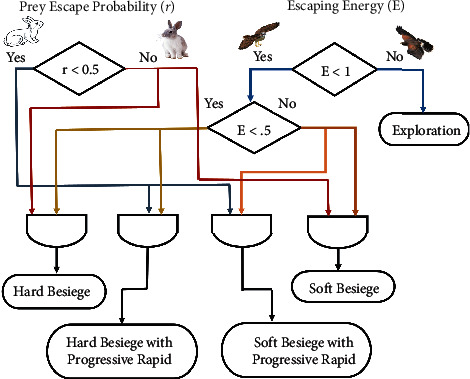
The different HHO possibilities in the exploitation stage [46].

In this stage, Harris Hawks move around the prey on the basis of calculated energy from different directions. The hawks' movement around the prey is considered as desired possible solution and the best possible solution is the position when hawks are closest to the prey. Harris Hawk attacking on the prey and running away from the prey are considered as two main behaviors of this stage. Harris Hawks attack on prey in a behavior which is known as surprise pounce. There are four different strategies proposed in HHO, depending upon escaping activities of prey and chasing style of hawks, namely soft and hard besiege, and soft besiege and hard besiege with progressive rapid dives. The four different strategies proposed in HHO can be utilized on the basis of two parameter, *E* escaping energy of the prey, and *r* probability of escaping of the prey. The values that lie between the range from 0 to 1 is known as probability of *r*. E energy value lies between the range −1 to 1. The possibilities of escaping energy *E* and probability of *r* are expressed in Figure 4. If *r*〈0.5, we can say that the prey has chances of escape, and if *r* ≥ 0.5, then the prey cannot escape. If |*E*| ≥ 0.5, then prey has no more energy to escape, but if |*E*| ≥ 0.5, then prey has enough energy to escape.

#### 3.7.4. Soft Besiege

This strategy is applied when prey has enough energy to escape *r* ≥ 0.5 and |*E*| ≥ 0.5. Prey can try to escape, but at the end, it cannot. Harris Hawks make softly besiege around the prey that makes the prey tired and at that time hawks implement the surprise pounce. This method is expressed as

(4) $$\begin{array}{c} Z\left(t+1\right)=\Delta Z\left(t\right)-E\left|JZ_{\text{prey}}\left(t\right)-Z\left(t\right)\right|, \end{array}$$

(5) $$\begin{array}{c} \Delta Z\left(t\right)=Z_{\text{prey}}\left(t\right)-Z\left(t\right). \end{array}$$

In equation (5), where Δ*Z*(*t*) signifies the position vector of the prey and the present location of hawk in the iteration *t*, *r*_5_ is the random value between 0 and 1 and *J*=2(1 − *r*_5_) represents escaping procedure of the prey. The *J* value arbitrarily changing in each iteration to mimic the behavior of prey movement.

*Hard Besiege:* this strategy is useful when prey has insufficient escaping energy and extremely exhausted *r* ≥ 0.5 and |*E*|〈0.5 . In this situation, prey totally tired; hawks make the circle around the prey and implement the surprise pounce. This strategy is expressed by

(6) $$\begin{array}{c} Z\left(t+1\right)=Z_{\text{prey}}\left(t\right)-E|\Delta Z\left(t\right)|. \end{array}$$

#### 3.7.5. Soft Besiege with Progressive Rapid Dives

This technique is useful when |*E*| ≥ 0.5 and *r*〈0.5. In this situation, prey has sufficient energy to escape and hawks perform soft besiege beforehand making surprise pounce. Hawks moves in a way, in which they choose the possible steps in the direction of the prey and also consider the significance of their possible next step in the direction of prey. If this step is correctly applied, then they use the equation (7), to modernize their recent position. If it is not correctly applied, then they use LF approach to attack on the prey in rapid dives by using equation (8).

(7) $$\begin{array}{c} Y=Z_{\text{prey}}\left(t\right)-E|JZ_{\text{prey}}\left(t\right)-Z\left(t\right)|, \end{array}$$

(8) $$\begin{array}{c} X=Y+S\times LF\left(D\right). \end{array}$$

Where dimension of the define problem is calculated by *D*, *S* is a random vector of size 1 × *D* and LF defines as levy flight function shown in equation (9). Where *u* and *v* are random numbers among 0 and 1 and *β* is the constant value set to 1.5. *σ* evaluated by using equation (9). The final step in which position of the hawk is updated, then in this situation soft besiege with progressive dives is calculated by equation (10).

(9) $$\begin{array}{c} LF\left(x\right)=0.01\times \frac{u\times \sigma }{{\left|v\right|}^{1/\beta }}, \end{array}$$

(10) $$\begin{array}{c} Z\left(t+1\right)=\left\{\begin{array}{c} X,\;\text{if}\;LF\left(X\right)\langle )LF\left(Z\left(t\right)\right), \end{array}\right) \end{array}$$

where fitness function is indicated by *f* and *Y* and *X* obtained by equations (7) and (8)

#### 3.7.6. Hard Besiege with Progressive Rapid Dives

This strategy is useful when |*E*|〈0.5 and *r*〈0.5. In this situation, prey has not enough energy to escape, and hawks perform hard besiege before making surprise pounce. This step is similar to the technique hawks use in soft besiege with progressive rapid dives. In this case, hawks consider the minimum distance between their average position and the prey's position. The following equation (11) is used in hard besiege:

(11) $$\begin{array}{c} Z\left(t+1\right)=\left\{\begin{array}{c} Y,\;\text{if}\;LF\left(Y\right)\langle )LF\left(Z\left(t\right)\right),\\ X,\;\text{if}\;LF\left(Z\right)\langle )LF\left(Z\left(t\right)\right). \end{array}\right) \end{array}$$

**Figure 5:**
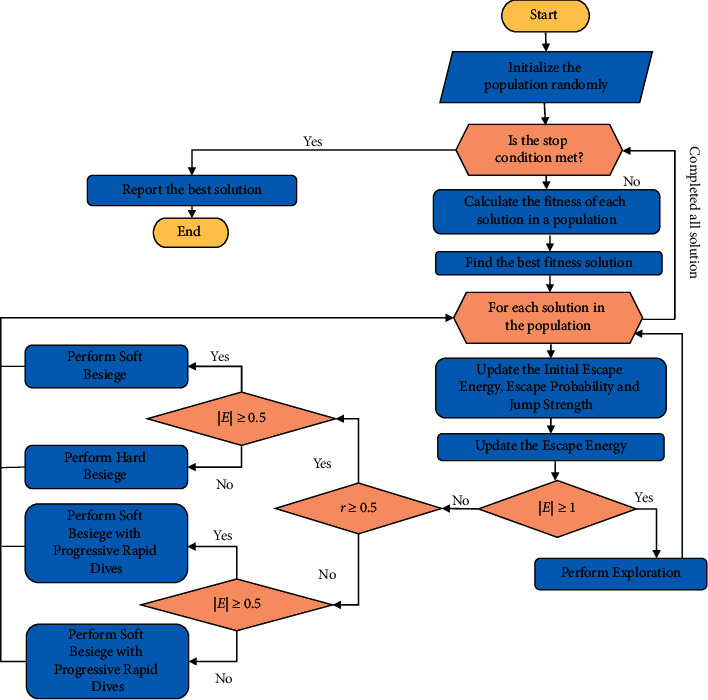
Flow chart of HHO.

The values of *Y* and *X* are obtained from the equations (12) and (13):

(12) $$\begin{array}{c} Y=Z_{\text{prey}}\left(t\right)-E\left|JZ_{\text{prey}}\left(t\right)-Z_{m}\left(t\right)\right|, \end{array}$$

(13) $$\begin{array}{c} X=Y+S\times LF\left(D\right), \end{array}$$

where *Z*_*m*_(*t*) is calculated from equation (2). The flowchart of the HHO is given in Figure 5.

### 3.8. Hybrid Approach of CNN-HHO

The motivation behind the proposed approach is that the deep neural network model works like a human-brain for processing the data and creates patterns for making the decision. Harris hawk optimizing algorithm recently developed the nature-inspired algorithm based on the hunting behavior of the hawks, and if we study the behaviors of Harris Hawk, they wait, observe, and monitor the prey with their powerful eyes and choose the best candidate solution for attack on the prey, where the prey has no chance to escape. Due to its ability to coverage quickly, when compared to other approach, HHO identifies the optimal solution in complex problem. HHO identifies the CNN optimum parameter fastly because it hardly gets stuck in local minima and enhances the model performance. We propose a hybrid approach of CNN-HHO in which we use Harris Hawk optimizing algorithm (HHO) for optimizing the CNN hyperparameters. Best hyperparameter and best performance of the model in terms of evaluation metrics are achieved from the proposed approach.

We trained CNN baseline-model with healthcare dataset, and for optimizing the hyperparameters of CNN, the algorithm Harris Hawk optimization is implemented from the opensource Mealpy python library [49]. In Algorithm 1, main phases of the proposed approach are outlined. We use two attributes of bug report dataset summary and severity of the bug. *X* is considered as summary, and *Y* is considered as target value. The data is split into testing and training ratio, *N* is the population size, and the total number of iterations “*T*.” Hyperparameters optimize through these iterations by using HHO. The hyperparameters that are optimized for improving the model performance are optimizer parameter *O*_*m*_, activation function *A*_*f*_, learning rate *L*_*r*_, batch size *B*_*s*_, and Kernel initializer *KB*_*n*_. The following hyperparameters selected value for optimization are shown in Table 4. The dataset is divided into training and testing ratio by using 10-fold cross validation.

Training dataset is used for training the classifier, and testing dataset is used for evaluating the performance of the model. First initial population is generated, and N defines the number of solutions. We use five hyperparameters for optimization, so solution size is 5. Number of solutions *N* and hyperparameter are considered as population matrix (*N*, 5). For hyperparameters lower bound and upper bound value range, fitness function is calculated. The detail of fitness value calculation is expressed in Algorithm 2. Best solution is mapped to hyperparameters. The CNN model is executed for evaluating the performance on optimized hyperparameters in the given value of epochs. We are working with binary classification problem, so for calculating the performance of model, we have used following evaluation metrics accuracy 14, precision 15, recall 16, and F1-measure 17. To estimate the accuracy of a model, calculate the ratio of true positive, false positive, false negative, and true negative.

(14) $$\begin{array}{c} \text{Accuracy}=\frac{TP+TN}{TP+TN+FP+FN},\\ \text{Precision}=\frac{TP}{TP+FP},\\ \text{Recall}=\frac{TP}{TN+FN},\\ F1-\text{measure}=\frac{2\times \text{Precision}+\text{Recall}}{\text{Precision}+\text{Recall}}. \end{array}$$

After evaluating the results of the five given metrics, it is important to map them into a single fitness value and for that we use the weighted sum method (WSM). We use the WSM in which we get the weight of each value and multiply with the given percentage, and then add all for calculating the fitness value. Fitness value is calculated by

(15) $$\begin{array}{c} \text{Fitness}=\left(0.25\times \text{Accuracy}+0.25\times \text{Precision}+0.25\times \text{Recall}+0.25\times F1-\text{measure}\right)\times 100\%. \end{array}$$

When fitness value is achieved for the given population, then we update the population for next iteration. The HHO is used from mealpy library, and detail working of the Harris Hawk is discussed above.

## 4. Experimental Results and Findings

This section evaluates the performance of the proposed model and baseline CNN model. The proposed hybrid approach is applied to healthcare bug report dataset. The convolutional neural network algorithm with Harris Hawk optimizing algorithm as a hyperparameter optimizing technique is implemented. In baseline CNN, the value of every parameter chooses manually and chooses basis on the best result. Number of convolution layers, number of dense layers, activation function at each layer, activation function at the output layer, number of epochs, batch size, number of pool layers, loss function, learning rate, optimizers etc. are the parameters of the convolutional neural network model. Manually identifying the right value for the parameters of the CNN model is a crucial task. Training time and the burden of the CNN model reduce by choosing an optimal value and for choosing optimal for the given parameters, Harris Hawk Algorithm (HHO) is used [50].

We have executed the CNN-HHO model on 10 optimization iteration for getting the best hyperparameters with the given dataset. The iterations are run with 10 epochs' value and 20 population size. In Table 5, we applied healthcare datasets to the proposed approach for getting the best hyperparameters. 32 batch size, Adagrad optimizer parameter, ReLU activation function, 0.03 learning rate, and uniform kernel initializer are the best hyperparameters with given dataset.

The accuracy, precision, recall, and f1-measure value are found on the basis of these selected best hyperparameters, and best results are written in the Table 6. Hybrid approach gives better results corresponding to best hyperparameters in terms of accuracy 96.21%, precision 88.06%, recall 92.54%, and f1-meaures 94.68%. After calculating the values of evaluation metrics, we calculated the fitness value of the proposed model by using WSM formula and performance in terms of WSM 92.86%. The results of the proposed approach are compared with the baseline CNN and are expressed in Table 6.

**Figure 6:**
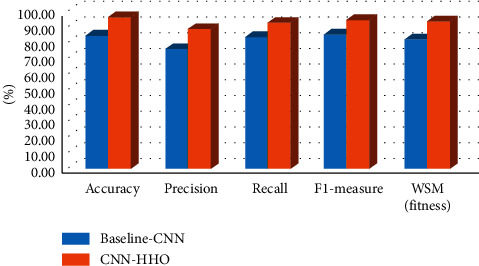
Comparison of the hybrid proposed approach with baseline-CNN model.

In Figure 6, we compared our proposed approach with baseline CNN model with the same dataset. The results clearly indicate that the proposed approach i.e., hyperparameter optimization of CNN with HHO performed better as compared to baseline CNN. The fitness value of the proposed model increased with 10.36% from baseline-CNN model.

### 4.1. Finding and Discussion

The experiment demonstrates that proposed hybrid approach for hyper-parameter optimization performed well on health care dataset. We used four metrics i.e., accuracy, precision, recall, and F1-measure for model performance evaluation. We have used WSM for calculating fitness value function. The proposed model gives best results as compared to the baseline-CNN.

**RQ 1**: What is the impact of the proposed approach of optimizing hyperparameter of the convolutional neural network algorithm on the accuracy of bug severity prediction?

The hybrid approach CNN-HHO is proposed for checking the impact of optimizing hyperparameters of the CNN algorithm on the accuracy. For optimizing the CNN hyperparameter, various techniques can be used such as grid search, Gradient Descent, and Meta-heuristic algorithm. We have used metaheuristic HHO for optimizing the hyperparameters of CNN. We choose five hyperparameters with their values of CNN such as optimizer parameters, activation function, batch size, learning rate, and Kernel initializers for optimizing with HHO on healthcare dataset. We are working with binary classification problem. The proposed model performed on HHO with 10 epochs' value and 20 population size. Best hyperparameter of model with corresponding best accuracy value is achieved. Batch zize with a value of 32, ReLU is the best activation function, Adagrad was the best optimizer parameter, learning rate with the value of 0.03, and uniform was the best Kernel initializer.

### 4.2. Threats to Validity

In this experiment, we find the different threats to validity that can impact on the performance of the proposed approach. These threats to validity are explained below.

#### 4.2.1. Internal Validity

The internal threats to validity are considered, as the Harris Hawk Optimization (HHO) algorithm is used for optimizing the hyperparameter of CNN in the experiment. For the prediction of bug severity, other hyperparameter optimization techniques can be used such as random search, grid search, gradient descent optimization techniques, and other metaheuristic algorithms such as PSO, GA, and GWO. Performance of the proposed approach can vary if different hyperparameter optimizing techniques are applied. In addition, for bug severity prediction, we just worked with CNN for achieving result because with textual dataset, CNN gives the best result. In spite of that, more models such as machine learning and deep learning classifiers can be used.

#### 4.2.2. External Validity

The external threats to validity depends on the quality of the dataset. The proposed approach implements on healthcare bug report dataset. In case if a different dataset is chosen, such as opensource projects and closed source projects, then the results of the approach may vary.

#### 4.2.3. Construct Validity

The construct validity is the selection of the evaluation metrics that evaluate the performance of the proposed approach. We have used accuracy, precision, recall, and f1-measure that are mostly used as evaluation metrics [6, 13]. We have worked with these metrics, and results are promising, if we work with different evaluation metrics like AUC, then results can vary.

## 5. Conclusion

Bugs with different severity levels are reported by users and testers, and it is very essential to solve the bugs on time by using the severity attribute. IoT devices based on healthcare support systems used for the treatment of Alzheimer's patients. These devices must be free of bugs because severe bugs can have severe consequences on the health condition of Alzheimer's patients. In this study, we have proposed a hybrid approach for the classification of bug severity problem that are based on CNN and HHO algorithms, with optimized hyperparameter of the CNN model with HHO. Healthcare dataset is used to validate the performance of the model. First we preprocessed the bug report dataset; second, we performed feature extraction for CNN embedding layer, and in the end, hyperparameter optimization is used for the HHO Algorithm. Batch size, learning rate, optimizer parameter, activation function, and Kernel initializer are used as hyperparameter for optimization. The hybrid CNN-HHO approach gave excellent results with the best hyperparameters. We used four metrics for evaluation of the performance of model accuracy, precision, recall, and F1-Measure. Fitness value is calculated by using WSM, and performance is achieved with 92.86% on the given dataset. We also compared our proposed model with the baseline CNN model. The accuracy of our model with value 96.21% is better as compared to another state-of-the-art technique. Future work can use different optimizing algorithms such as GA, PSO, and GWO for improving the model performance and achieving the best hyperparameters of the model; large dataset can be used for checking the proposed hybrid CNN-HHO model, to improve the accuracy of bug severity classification problem, and other DL approaches such as LSTM, RNN etc. can be used with the optimizing algorithm for bug severity prediction problem.

## Figures and Tables

**Algorithm 1:**
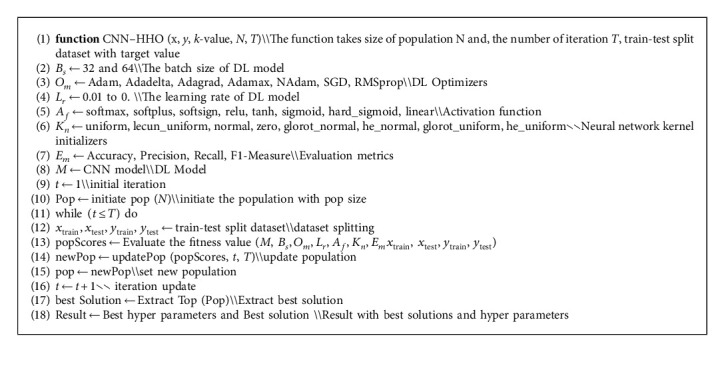
Pseudocode of the proposed approach hybrid CNN-HHO.

**Algorithm 2:**
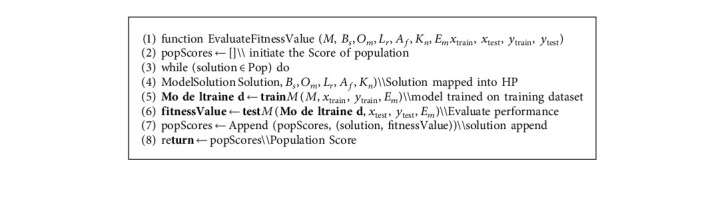
Pseudocode of the fitness value calculation.

**Table 1:** Table of abbreviations.

Abbreviation	Full form
Adadelta	Adaptive delta
Adagrad	Adaptive gradient
Adam	Adaptive moment estimation
AdaMax	Adaptive max-pooling
AdaBoost	Adaptive boosting
ACO	Ant colony optimizer
AUC	Area under cover
BTS	Bug tracking system
CMT	Class membership information of a term
CNN	Convolution neural network
CNRFB	Convolutional neural network and ransom forest with boosting classifier
DL	Deep learning
J48	Decision tree
XGBoost	Extreme gradient boosting
GA	Genetic algorithm
GWO	Gray wolf optimizer
HHO	Harris Hawk optimization
IoT	Internet of Things
KNN	K-nearest neighbor
LF	Levy flight
LSTM	Long short term memory
LMT	Logistic model trees
ML	Machine learning
MIoT	Medical in internet of things
NB	Naïve Bayes
MRIs	Magnetic resonance images
NLP	Natural language preprocessing
NLTK	Natural language toolkit
OS	Operating system
PSO	Particle swarm optimization
RLU	Rectified linear unit
RNG	Relative neighbor graph
RF	Random forest
SMOTE	Synthetic minority oversampling technique
SVM	Support vector machine
WSM	Weighted sum method

**Table 2:** Bugreport preprocessing example.

Original statement	Export from the UI should create compressed repositories
Tokenization	“Export,” “from,” “the,” “ui,” “should,” “create,” “compressed,” “repositories”
Stopword removal	“Export,” “ui,” “create,” “compressed,” “repositories”
Lemmatization	“Export,” “ui,” “create,” “compressed,” “repository”

**Table 3:** Explanation of symbols used in the HHO algorithm [48].

Symbols	Description
Z, *Z*_*i*_	Position vector of the hawks in the iteration of *i*-th hawk
*Z*_prey_	Position of the prey
*Z*_rand_	Position of random hawk
*Z*_*m*_	Average position of the hawks
*E*, *E*_*o*_	Escaping energy of prey, initial state of energy
*N*, *t*, *T*	Swarm size, iteration counter, maximum number of iterations
*L*_*B*_, *U*_*B*_, *D*	Variable values (lower and upper bound), dimension
*r*_1_, *r*_2_, *r*_3_, *r*_4_, *r*_5_, *p*	Random number in the range (0, 1)

**Table 4:** Details of hyperparameter of CNN classifier that are optimized.

Hyper-parameters	Values
Activation function	Softmax, Softplus, Softsign, ReLU, tanh, sigmoid, hard_sigmoid, linear
Optimizer parameters	Adam, Adadelta, Adagrad, Adamax, NAdam, SGD, RMSprop
Kernel initializers	Uniform, lecun_uniform, normal, zero, glorot_normal, he_normal, glorot_uniform, he_uniform
Learning rate	0.01 to 0.5
Batch size	32 and 64

**Table 5:** Selected hyperparameters of hybrid approach CNN-HHO on healthcare dataset.

Dataset	Hyperparameter	Selected values
Healthcare	Activation function	ReLU
	Optimizer parameters	Adagrad
	Kernel initializers	Uniform
	Learning rate	0.03
	Batch size	32

**Table 6:** Performance comparison of baseline-CNN and hybrid CNN-HHO model on healthcare dataset.

Models	Accuracy (%)	Precision (%)	Recall (%)	F1-measure (%)	WSM (fitness value)
Baseline-CNN	84.58	75.98	83.93	85.52	82.50%
CNN-HHO	96.21	88.06	92.54	94.68	92.86%

## Data Availability

The [IoT Medical Device's Dataset for Bug Sever- ity prediction] data used to support the findings of this study have been deposited in the [Kaggle] repos- itory ([https://www.kaggle.com/datasets/iqrayousaf/iot- medical-devices-dataset]).
